# microRNA-9 Inhibits Vulnerable Plaque Formation and Vascular Remodeling *via* Suppression of the SDC2-Dependent FAK/ERK Signaling Pathway in Mice With Atherosclerosis

**DOI:** 10.3389/fphys.2020.00804

**Published:** 2020-07-16

**Authors:** Ruihong Zhang, Beibei Song, Xiaojian Hong, Zhiyuan Shen, Li Sui, Siyu Wang

**Affiliations:** ^1^Department of Cardiology, The Fourth Affiliated Hospital of Harbin Medical University, Harbin, China; ^2^Department of Emergency, The First Affiliated Hospital of Harbin Medical University, Harbin, China; ^3^Department of Medical Oncology, The First Affiliated Hospital of Harbin Medical University, Harbin, China

**Keywords:** microRNA-9, SDC2, FAK/ERK signaling pathway, atherosclerosis, acute coronary syndrome

## Abstract

microRNAs (miRNAs or miRs) play important roles in modulating the occurrence and progression of atherosclerosis and acute coronary syndrome (ACS). Herein, this study aimed to investigate the possible role of miR-9 in the development of atherosclerosis. Initially, the differentially expressed genes associated with ACS were screened and miRNAs that regulate syndecan-2 (SDC2) were predicted using microarray analysis. Furthermore, the biological functions of miR-9 and SDC2 on aortic plaque area, proliferation of collagen fibers, Mac-3-labeled macrophages, inflammatory response, and levels of the focal adhesion kinase/extracellular signal-regulated kinase (FAK/ERK) signaling pathway-related proteins in atherosclerosis were evaluated after ectopic miR-9 expression or SDC2 depletion in ACS mice using oil red O staining, Masson’s trichrome staining, immunohistochemistry, and Western blot analysis, respectively. SDC2 was highly-expressed, while miR-9 was poorly-expressed in atherosclerosis. Additionally, miR-9 targeted SDC2 and negatively-regulated its expression. Up-regulation of miR-9 reduced aortic plaque area, the proliferation of collagen fibers, Mac-3-labeled macrophages and levels of IL-6, IL-1β, and TNF-α by suppressing SDC2 and the FAK/ERK signaling pathway, thereby ameliorating atherosclerosis in ACS mice. In conclusion, the current study provides evidence that miR-9 retards atherosclerosis by repressing SDC2 and the FAK/ERK signaling pathway, highlighting a new theoretical basis for the treatment of atherosclerosis.

## Introduction

Acute coronary syndrome (ACS) is the most fatal form of ischemic heart disease, including ST-segment elevation myocardial infarction (STEMI), non-STEMI, and unstable angina (Falk et al., 2013). The main clinical presentations in ACS consist of acute myocardial infarction, unstable angina (Wang and Jin, 2020), chest pain, sweating, and nausea (Khan et al., 2013). Advanced atherosclerosis with thrombus is one of the main characteristics of ACS (Libby et al., 2019). Furthermore, it is reported that as a chronic inflammatory disorder in the arterial wall, atherosclerosis correlates to vascular smooth muscle cell dedifferentiation, migration and proliferation, endothelial dysfunction, inflammation, foam cell formation, and insulin resistance (Martinez et al., 2018; Yu et al., 2018). Also, vascular endothelial cell injury is one of the leading causes of plaque erosion and thrombosis formation, which ultimately contributes to the incidence and progression of ACS and atherosclerosis (Zhu et al., 2011). As previously reported, atherosclerosis is one of the principle causes of morbidity and mortality (Wang et al., 2018). Therefore, it is critical to explore the molecular mechanism of underlying atherosclerosis for exploration of novel target for treatment of atherosclerosis.

MicroRNAs (miRNAs or miRs) are small, non-coding RNA molecules that regulate gene expression by binding to the 3’untranslated region (3’UTR) of target mRNAs, exerting important roles in numerous pathological conditions, including cardiovascular disease (Das and Halushka, 2015). Existing literature also indicates that multiple miRNAs, such as miR-1, miR-21, miR-499, miR-133a, miR-146a, miR-208a, miR-92a, and miR-126-3p, can potentially exert diagnostic and prognostic performance in coronary artery diseases and ACS (Shirvani Samani and Meder, 2016; Santovito and Weber, 2017). Another such miRNA, the miR-9 is reported to repress inflammation in atherosclerosis (Wang et al., 2017), while the specific mechanisms of miR-9 in ACS have not been reported. Web-available microarray analysis further indicated that Syndecan-2 (SDC2) is a potential target gene of miR-9. SDC2 is known to serve as a cell surface receptor which mediates adhesion-dependent signal transduction in the process of cell growth, migration, and differentiation (Huang et al., 2009). SDC2 has also been documented to exert particular effects on atherosclerotic plaque formation (Geeraert et al., 2007). In addition, a recent study revealed that SDC2 could promote the focal adhesion kinase (FAK)/extracellular signal-regulated kinase (ERK) signaling pathway by elevating the expression of matrix metalloproteinase (MMP)-7 (Jang et al., 2017). FAK, also known as protein tyrosine kinase 2 (PTK2), can significantly impact the regulation of cell migration and focal adhesion (Khosravi et al., 2019). Moreover, Zhang et al. (2012) demonstrated that activation of the ERK2 signaling pathway was associated with the progression of ACS, suggesting a potential target for the treatment of ACS. However, the effects of miR-9 targeting SDC2 *via* the FAK/ERK signaling pathway on atherosclerosis in ACS remain to be unclear. In the current study, we aimed to elucidate the role of the newly discovered miR-9 in atherosclerosis in ACS and its underlying mechanisms, which may help to provide a novel direction for treating atherosclerosis.

## Materials and Methods

### Ethics Statement

Written informed consents were obtained from all participants prior to enrollment. Study protocols were approved by the Ethics Committees of the Fourth Affiliated Hospital of Harbin Medical University and strictly adhered to the ethical principles for medical research involving human subjects of the *Declaration of Helsinki*. Animal experiments were performed strictly in accordance with the Guide for the Care and Use of Laboratory Animals published by the US National Institutes of Health. Animal experimentation protocols were approved by the Institutional Animal Care and Use Committee of the Fourth Affiliated Hospital of Harbin Medical University, and all efforts were made to minimize the suffering of the included animals.

### Study Subjects

A total of 86 patients who were diagnosed with ACS at the Fourth Affiliated Hospital of Harbin Medical University between March 2017 and September 2017 were enrolled in the current study. Peripheral blood samples were collected from all participants as the study subjects. An additional 86 healthy volunteers were also enrolled and their peripheral blood samples were obtained as the control. All patients underwent coronary angiography and iMap-intravascular ultrasound (IVUS) at the Fourth Affiliated Hospital of Harbin Medical University. Patients presenting with complete occlusion, restenosis after stent implantation, coronary artery bypass grafting, severe heart failure (New York Heart Association function at grade III or above), renal failure (creatinine clearance rate < 30 mL/min), hepatic insufficiency, infectious diseases, patches unsuitable for analysis, or insufficient clinical data were excluded from the study.

### Establishment of Atherosclerosis Mouse Models

A total of 50 male ApoE knockout (ApoE^–/–^) mice (aged 8 weeks, weighing 20 ± 2 *g*, C57BL/6 background) were purchased from the Experimental Animal Center of Peking University. Ten ApoE^–/–^ mice were fed with normal diet to serve as the control, while the remaining 40 ApoE^–/–^ mice were fed with high-fat and high-cholesterol atherogenic foods (containing 20.0% fat, 19.5% casein, and 0.5% cholesterol) serving as the high-fat diet (HFD) group. The HFD mice were then treated with short hairpin RNA-negative control (sh-NC) vector, sh-SDC2, agomir NC, and miR-9 agomir. The miR-9 agomir or agomir NC was dissolved in 0.2 mL normal saline and injected into the mice at a dose of 80 mg/kg/d *via* the tail vein once a day for ten consecutive days (Yu et al., 2020). Lentivirus carrying oe-SDC2 or sh-SDC2 was then injected into the mice at a dose of 1 × 10^7^ PFU/mouse *via* the tail vein, once every 2 days. The survival rate of atherosclerosis mouse models was calculated to be 85% (34/40). After feeding for 8 weeks, all mice were euthanized. Fresh whole blood samples were extracted before euthanasia and the mice were deprived of food but not water for 24 h before euthanasia. Finally, eight mice from each group were randomly selected for subsequent experimentation.

### Monocyte Isolation

A total of 40 mL blood was extracted from the carotid artery of patients with ACS and the plasma was removed. Peripheral blood mononuclear cells (PBMCs) were isolated using gradient separation on Histopaque-1.119. The obtained PBMCs were washed thrice in Ca^2+^/Mg^2+^-free phosphate-buffered saline (PBS) and counted. Next, the cells were diluted to a concentration of 5 × 10^7^ cells/mL with Ca^2+^/Mg^2+^-free PBS containing 0.5% BSA and 2 mM ethylenediaminetetraacetic acid (EDTA). Every 5 × 10^7^ cells were incubated with 100 μL CD14 microbeads at 8°C for 15 min. The cells were washed once and then re-suspended in 500 μL of Ca^2+^/Mg^2+^-free PBS containing 0.5% BSA and 2 mM EDTA. The suspension was applied to a MidiMACS Separator (Miltenyi, Bergisch Gladbach, Germany) for positive selection of the magnetically labeled CD14^+^ cells.

### RNA Isolation and Quantitation

Total RNA was extracted from PBMCs of patients with ACS and aortic tissues of ApoE^–/–^ mice using the TRIzol reagent (15596026, Invitrogen, Carlsbad, CA, United States) according to the manufacturer’s instructions. Next, the extracted total RNA was reverse-transcribed into complementary DNA (cDNA) according to the manufacturer’s instructions of PrimeScript RT reagent kits (RR047A, Takara Bio Inc., Otsu, Shiga, Japan) or Ncode TM miRNA First-Strand cDNA Synthesis Reverse transcription kits (Thermo Fisher Scientific, Rockford, IL, United States). The obtained cDNA was applied for subsequent reverse transcription-quantitative polymerase chain reaction (RT-qPCR) using the Fast SYBR Green PCR kit (Applied Biosystems, Carlsbad, CA, United States) on an ABI 7300 instrument (Applied Biosystems, Foster City, CA, United States). Three replicates were set in each group. U6 was regarded as the internal reference for miR-9, while glyceraldehyde-3-phosphate dehydrogenase (GAPDH) was regarded as the internal reference for SDC2. The expression ratio of the target gene between the experimental and control groups was calculated using the 2^–ΔΔCt^ method. The primer sequences are shown in Supplementary Table 1.

### Western Blot Analysis

Total protein was extracted from PBMCs of patients with ACS, aortic lysate from ApoE^–/–^ mice or transfected 293T cells using enhanced radio-immunoprecipitation assay lysis (BOSTER Biological Technology Co., Ltd., Wuhan, Hubei, China) supplemented with protease inhibitor. Protein concentration was determined using bicinchoninic acid kits (BOSTER Biological Technology Co. Ltd., Wuhan, Hubei, China). Next, 30 μg of the extracted protein was separated with 10% sodium dodecyl sulfate-polyacrylamide gel electrophoresis and transferred onto a polyvinylidene fluoride membrane (Millipore, Billerica, MA, United States). The membrane was then blocked with 5% bovine serum albumin for 2 h to block the non-specific binding, followed by incubation overnight at 4°C with the diluted primary rabbit antibodies against SDC2 (ab205884, dilution ratio of 1: 500), ERK1/2 (ab17942, dilution ratio of 1: 1000), phosphorylated (p)-ERK1/2 (ab214362, dilution ratio of 1: 500), FAK (ab40794, dilution ratio of 1: 2000), p-FAK (ab81298, dilution ratio of 1: 1000), and GAPDH (ab18602, dilution ratio of 1: 5000). All the above-mentioned antibodies were purchased from Abcam Inc. (Cambridge, United Kingdom). After 3 rinses with phosphate-buffered saline Tween-20 (PBST), the membrane was re-probed with horseradish peroxidase (HRP)-labeled secondary goat anti-rabbit immunoglobulin G (IgG; ab205719; dilution ratio of 1: 2000, Abcam Inc., Cambridge, United Kingdom) for 1 h, followed by three rinses with PBST. After that, the immunocomplexes on the membrane were visualized using enhanced chemiluminescence (EMD, Millipore, Billerica, MA, United States). Thereafter, the Image J software was employed for the quantitative analysis of protein with GAPDH as the internal reference.

### Oil Red O Staining

Phosphate-buffered sali was slowly perfused into the left ventricle of mice after euthanasia. The aorta was separated, and whole aorta specimens were then placed in 60% isopropanol for 10 min, soaked in oil red O solution for 3–4 h, and differentiated six to seven times with 60% isopropanol. Next, the samples were photographed under a light microscope. The total areas of aorta and lipid striation were analyzed using the Image J software, and the percentage of lipid striation in total aorta area was calculated.

### Hematoxylin-Eosin (HE) Staining

Phosphate-buffered sali was slowly perfused into the left ventricle of mice after euthanasia. Next, the aortic brachiocephalic division was separated, fixed with 4% paraformaldehyde, paraffin-embedded and sectioned. The paraffin-embedded sections were then dewaxed, immersed in xylene I, xylene II, and gradient alcohol for 5 min, respectively, and washed with water for 2 min. The sections were stained with hematoxylin for 8 min, differentiated with 1% hydrochloric acid alcohol for 5 s, and treated with 0.25% ammonia water for 1 min. After staining with 1% eosin for 30 s, the samples were washed three times with water, followed by immersion in gradient alcohol, xylene I, and xylene II for 3 min, respectively. When the xylene on the glass dried completely, the clean cover slide was sealed with neutral gum. Pathological changes in blood vessels were observed under a microscope (CX41, Olympus, Tokyo, Japan). An image analysis system was adopted to analyze the indicators of vascular remodeling (VR), including medium wall thickness (MT), lumen diameter (LD), and medium wall thickness/lumen diameter (MT/LD). Three visual fields were selected to calculate the mean value.

### Masson’s Trichrome Staining

Mice were anesthetized with 2% sodium pentobarbital (50 mg/kg), with the thoracic aorta isolated. After being fixed with 4% paraformaldehyde, the samples were dehydrated, cleared, waxed, paraffin-embedded, sliced, stained with sirius picric acid red, and counter-stained with hematoxylin (CAS No. PT003, Shanghai Bogoo Biotechnology Co., Ltd., Shanghai, China). Next, the specimens were photographed under a polarized light microscope (XPT-480, Shanghai Zhongheng Instruments Co., Ltd., Shanghai, China) and analyzed using the Image-Pro Plus 6.0 software (Media Cybernetics Inc., Bethesda, MD, United States). Five visual fields were randomly selected from each slice to analyze the myocardial collagen volume fraction (CVF), and myocardial CVF (%) = collagen area of aorta/measured visual field area × 100% (Li et al., 2015).

### Immunohistochemistry

The paraffin-embedded sections were dried in an oven at 60°C for 30 min, followed by conventional dewaxing and hydration with xylene I, xylene II, and gradient alcohol for 5 min, respectively. After undergoing antigen retrieval in a microwave with 1 mM Tris–ethylene diamine tetraacetic acid (pH 8.0) and being allowed to cool down to room temperature, the sections were rinsed thrice with PBS (each time 5 min), treated with 3% H_2_O_2_ at room temperature for 10 min to block the endogenous peroxidase activity, and rinsed twice with PBS (each time 5 min). Subsequently, the sections were incubated with the primary antibody, rabbit anti-mouse against Mac-3 polyclonal antibody (ab9829, dilution ratio of 15 μg/mL, Abcam Inc., Cambridge, United States) or rabbit polyclonal antibody against SDC2 (ab205884, dilution ratio of 1: 200, Abcam Inc., Cambridge, United Kingdom) at 4°C overnight. The following day, the specimens were rinsed thrice with 0.1% PBST (each time 5 min), and then incubated with polymer reinforcer (PV-9000, Beijing ZSGB Co., Ltd., Beijing, China) at room temperature for 20 min, followed by 3 rinses with 0.1% PBST (each time 5 min), and another incubation with enzyme-labeled anti-mouse/rabbit polymer (PV-9000, Beijing ZSGB Co., Ltd., Beijing, China) at room temperature for 30 min. After three rinses with 0.1% PBST times (each time 5 min), the sections were developed with diaminobenzidine for 5 min, which was halted by washing with distilled water. Next, the sections were counterstained with hematoxylin, differentiated, and blued in water. Finally, the sections were dehydrated, cleared, and sealed with neutral gum. The sealed sections were observed and photographed under a microscope (CX41, Olympus, Tokyo, Japan).

### Enzyme-Linked Immunosorbent Assay (ELISA)

Before euthanasia, fresh whole blood of mice was centrifuged at 500 *g* and 4°C for 30 min, and the supernatant was collected. Then the levels of serum interleukin (IL)-6 (SM-E60005), IL-1β (E0563), and tumor necrosis factor (TNF)-α (SM-E60220) were determined. All kits were purchased from Rapidbio (Hillsborough, NJ, United States). The experiment was conducted strictly according to the manufacturer’s instructions of the kit. The optical density (OD) value of each well was measured at a wavelength of 450 nm with a microplate reader (Thermo Fisher Scientific, Waltham, MA, United States), and the relative expression of each inflammatory factor was calculated.

### Dual-Luciferase Reporter Gene Assay

The HEK293T cells (American Type Culture Collection, Manassas, VA, United States) at the logarithmic phase of growth were seeded in 6-well plates at a density of 2 × 10^5^ cells/well. After cell adherence, transfection was carried out according to the aforementioned method. After successful transfection, cells were cultured for 48 h and harvested. The SDC2-3’UTR-wild type (wt) and SDC2-3’UTR-mutant (mut) sequences were cloned into pGL3 vectors *via* restriction endonuclease, and then co-transfected with mimic NC or miR-9 mimic into HEK293T cells. Lipofectamine 2000 reagents (Invitrogen, Carlsbad, California, United States) were used for cell transfection. The luciferase activity was determined using the Dual-Luciferase Reporter Gene Assay kits (D0010, Beijing Solarbio Science & Technology Co., Ltd., Beijing, China) according to the manufacturer’s instructions on a Glomax20/20 Luminometer (E5311, Shanxi Zhongmei Biotechnology Co., Ltd., Xi’an, China). The relative luciferase activity was calculated as ratio of firefly luciferase activity/renilla luciferase activity.

### Statistical Analysis

All data analyses were performed using the SPSS 21.0 software (IBM Corp. Armonk, NY, United States). All measurement data conforming to normal distribution and homogeneity of variance were expressed as mean ± standard deviation. Comparisons within the group were analyzed using paired *t*-test, while comparisons between two groups were analyzed using the unpaired *t*-test. Comparisons among multiple groups were tested by one-way analysis of variance (ANOVA), followed by Tukey’s test. Comparisons of data at different time points were tested by repeated measures ANOVA, followed by Bonferroni corrections. A value of *p* < 0.05 was considered statistically significant.

## Results

### SDC2 Was Up-Regulated in PBMCs of Patients With ACS and Aortic Lysate From HFD-Fed ApoE^–/–^ Mice

**FIGURE 1:**
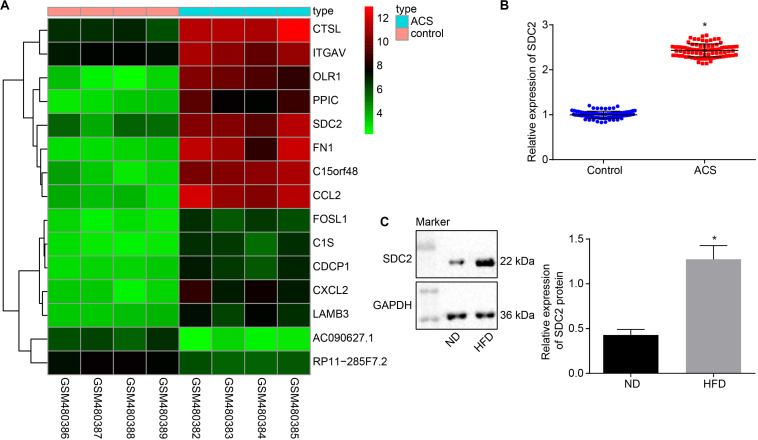
SDC2 is highly-expressed in atherosclerosis. **(A)** A heatmap of the top 15 DEGs in ACS-related microarray data GSE19339. Abscissa indicates sample number and the ordinate indicates DEGs. Histogram in the upper right refers to color gradation and small squares in the figure represent the expression of a gene in one sample; **(B)** SDC2 expression patterns determined by RT-qPCR in the PBMCs isolated from patients with ACS (*n* = 86) and healthy individuals (*n* = 86), normalized to GAPDH; *p < 0.05 *vs.* healthy individuals; and **(C)** Representative Western blots of SDC2 protein and its quantitation in the aortic lysate of HFD-fed ApoE^–/–^ mice (*n* = 40) and ND-fed ApoE^–/–^ mice (*n* = 10), normalized to GAPDH; ND refers to ApoE^–/–^ mice fed with normal diet and HFD refers to ApoE^–/–^ mice fed with high-fat diet. *p < 0.05 *vs.* ND-fed ApoE^–/–^ mice. All data were measurement data and expressed as mean ± standard deviation. Comparisons in panel **(B)** were analyzed using unpaired *t*-test, while comparisons in panel C were analyzed using paired *t*-test.

Firstly, the ACS-related microarray dataset GSE19339 was retrieved from the Gene Expression Omnibus (GEO) database^1^ and analyzed with the limma package of R language^2^ to identify the differentially expressed genes (DEGs). Subsequently, a heat map of the top 15 DEGs was plotted using the heatmap package^3^, which revealed that the expression of SDC2 was augmented in ACS (Figure 1A). A previous literature reported the direct correlation of high SDC expression in pig blood monocytes with coronary atherosclerosis (Geeraert et al., 2007). Therefore, in order to further verify the expression of SDC2 in ACS, RT-qPCR was performed to determine SDC2 expression in the PBMCs isolated from patients with ACS. The findings suggested that SDC2 level was elevated in PBMCs from patients with ACS (*p* < 0.05; Figure 1B). Moreover, Western blot analysis revealed that the aortic lysate from HFD-fed ApoE^–/–^ mice presented with higher SDC2 expression relative to ND-fed ApoE^–/–^ mice (*p* < 0.05; Figure 1C). These findings methodically revealed that the SDC2 gene was highly-expressed in ACS.

### Silencing of SDC2 Suppresses Atherosclerosis *via* Inhibition of the FAK/ERK Signaling Pathway in HFD-Fed Mice

**FIGURE 2:**
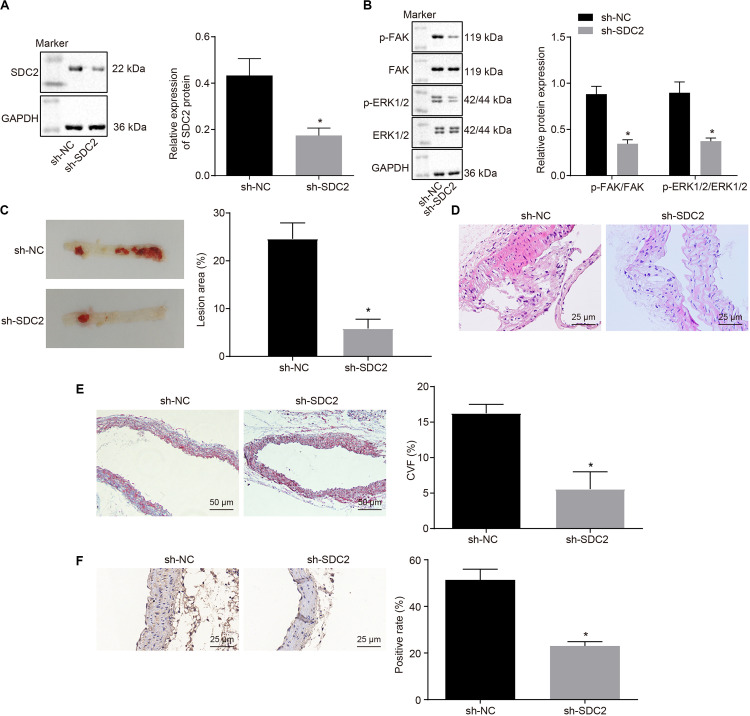
Down-regulation of SDC2 inhibits the FAK/ERK signaling pathway, and thus relieves atherosclerosis in mice. **(A)** Representative Western blots of SDC2 protein and its quantitation in the aortic lysate of mice after depletion of SDC2, normalized to GAPDH; **(B)** Representative Western blots of FAK, and ERK1/2 proteins and their quantitation in the aortic lysate of mice after depletion of SDC2, normalized to GAPDH; **(C)** Aortic plaque area determined using oil red O staining in mice after depletion of SDC2; **(D)** Lesion degree of aorta determined using HE staining in mice after depletion of SDC2 (400×); **(E)** The proliferation of collagen fibers measured using Masson’s trichrome staining in the aorta of mice after depletion of SDC2 (200×); and **(F)** Mac-3-labeled macrophages surrounding the aorta measured using immunohistochemistry in mice after depletion of SDC2 (400×); *p < 0.05 *vs.* mice treated with sh-NC; *n* = 8 for mice upon each treatment. All data were measurement data and expressed as mean ± standard deviation. Comparisons were analyzed using the unpaired *t*-test. The experiment was repeated 3 times.

After uncovering the high expression of SDC2 in ACS, we next aimed to investigate the regulatory mechanism of SDC2 in atherosclerosis by silencing SDC2 in mice *via* lentivirus. Results of Western blot analysis demonstrated that mice treated with sh-SDC2 exhibited decreased SDC2 expression compared to mice treated with sh-NC (*p* < 0.05; Figure 2A). In addition, compared to mice treated with sh-NC, the expression of p-FAK/FAK, and p-ERK/ERK was found to be reduced in the aortic lysate obtained from mice treated with sh-SDC2 (both *p* < 0.05; Figure 2B). Oil red O staining illustrated that compared to mice treated with sh-NC, the mice treated with sh-SDC2 showed a notable reduction in aortic plaque area (*p* < 0.05; Figure 2C). Additionally, HE staining revealed hyperplasia, disordered arrangement and obvious loss of endothelial cells in mice treated with sh-NC. Meanwhile, the lesion degree was also relieved in mice treated with sh-SDC2 relative to mice treated with sh-NC (*p* < 0.05; Figure 2D). The indicators of VR showed that mice treated with sh-SDC2 demonstrated a notable decrease in MT and MT/LD and significant increase in LD compared to mice treated with sh-NC (*p* < 0.05; Table 1). Also, the findings of Masson’s trichrome staining, immunohistochemistry, and ELISA displayed that when compared to mice treated with sh-NC, mice treated with sh-SDC2 exhibited a significant reduction in the proliferation of aortic collagen fibers, vascular CVF, and macrophages (labeled by Mac-3 antibody surrounding the aorta of mice) in atherosclerotic plaque of aorta, as well as the levels of serum IL-6, IL-1β, and TNF-α (*p* < 0.05; Figures 2E,F and Table 2). These data indicated that the knockdown of SDC2 relieved atherosclerosis by inhibiting the FAK/ERK signaling pathway in HFD-fed mice.

**TABLE 1:** Comparisons of VR indicators after depletion of SDC2 in mice.

Groups	MT	LD	MT/LD (%)
sh-NC	71.34 ± 9.63	3.05 ± 0.97	25.82 ± 9.85
sh-SDC2	36.98 ± 4.21*	7.24 ± 0.89*	5.21 ± 1.06*

VR, vascular remodeling; MT, medium wall thickness; LD, lumen diameter; MT/LD, medium wall thickness/lumen diameter; NC, negative control; *p < 0.05 *vs.* mice treated with sh-NC; *n* = 8 for mice upon each treatment; All data were measurement data and expressed as mean ± standard deviation. Comparisons were analyzed using the unpaired *t*-test. The experiment was repeated 3 times.

**TABLE 2:** Levels of serum inflammatory factors of mice after depletion of SDC2.

Inflammatory factors (pg/mL)	IL-6 (pg/mL)	IL-1β (pg/mL)	TNF-α (pg/mL)
sh-NC	6.71 ± 0.92	12.61 ± 0.25	8.34 ± 0.18
sh-SDC2	3.24 ± 1.03*	6.34 ± 0.16*	4.68 ± 0.13*

IL, interleukin; TNF-α, tumor necrosis factor-α; NC, negative control; *p < 0.05 *vs.* mice treated with sh-NC; *n* = 8 for mice upon each treatment; All data were measurement data and expressed as mean ± standard deviation. Comparisons were analyzed using the unpaired *t*-test. The experiment was repeated 3 times.

### SDC2 Was a Target Gene of miR-9

**FIGURE 3:**
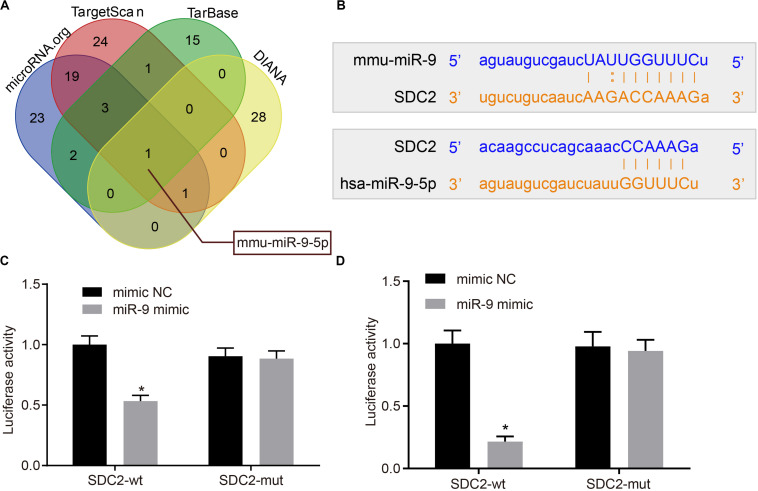
miR-9 targets and negatively-regulates SDC2. **(A)** Predicted upstream miRNAs of SDC2 by the DIANA, TarBase, TargetScan, and microRNA.org databases; **(B)** Putative miR-9 binding sites in the 3’UTR of SDC2 mRNA following bioinformatics analysis; **(C)** mmu-miR-9 binding with the 3’UTR of SDC2 mRNA confirmed by dual-luciferase reporter gene assay; and **(D)** hsa-miR-9 binding with the 3’UTR of SDC2 mRNA confirmed by dual-luciferase reporter gene assay. *p < 0.05 *vs*. cells treated with mimic NC; *n* = 8 for mice upon each treatment. All data were measurement data and expressed as mean ± standard deviation. Comparisons were analyzed using the unpaired *t*-test. The experiment was repeated 3 times.

Various databases included DIANA^4^, TarBase^5^, TargetScan^6^, and microRNA.org^7^ were employed to predict the miRNAs potentially regulating SDC2 in order to further explore the possible upstream regulatory mechanism of SDC2. Following Venn diagram analysis of the predicted miRNAs (Figure 3A), only miR-9 was found at the intersection, suggesting that miR-9 was likely to target SDC2. Bioinformatics analysis also indicated the presence of a specific binding site between SDC2 and miR-9 (Figure 3B). Subsequently, dual-luciferase reporter gene assay was performed, which further verified SDC2 as a target gene of miR-9. Compared with cells treated with mimic NC, the luciferase activity of SDC2-3’UTR-wt was decreased in cells following miR-9 mimic transfection (*p* < 0.05), but no difference was found in the luciferase activity of SDC2-3’UTR-mut (*p* > 0.05; Figures 3C,D). These findings revealed that SDC2 was indeed a target gene of miR-9, and miR-9 negatively-regulated SDC2.

### miR-9 Was Down-Regulated in PBMCs of Patients With ACS and Aortic Lysate From HFD-Fed ApoE^–/–^ Mice

**FIGURE 4:**
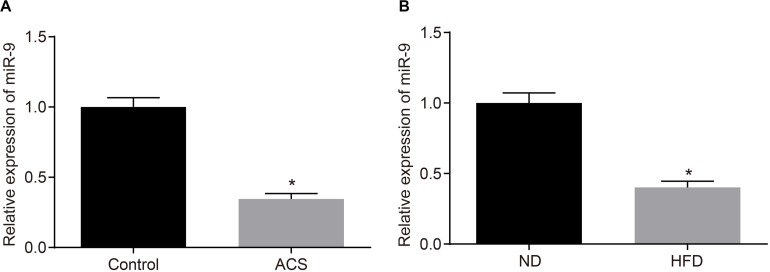
miR-9 is poorly-expressed in ACS. **(A)** miR-9 expression patterns determined by RT-qPCR in PBMCs isolated from patients with ACS (*n* = 86) and healthy individuals (*n* = 86), normalized to U6; *p < 0.05 *vs.* healthy individuals; **(B)** miR-9 expression patterns determined by RT-qPCR in the aortic lysate of HFD-fed ApoE^–/–^ mice (*n* = 40) and ND-fed ApoE^–/–^ mice (*n* = 10), normalized to U6. ND refers to ApoE^–/–^ mice fed with normal diet and HFD refers to ApoE^–/–^ mice fed with high-fat diet; *p < 0.05 *vs.* ND-fed ApoE^–/–^ mice. All data were measurement data and expressed as mean ± standard deviation. Comparisons were analyzed using the unpaired *t*-test.

Additionally, the miR-9 expression patterns in the PBMCs isolated from patients with ACS were determined with RT-qPCR to further elucidate the role of miR-9 in ACS. The findings suggested that miR-9 expression was reduced in the PBMCs from patients with ACS (*p* < 0.05; Figure 4A). Moreover, RT-qPCR revealed that compared to ND-fed ApoE^–/–^ mice, HFD-fed ApoE^–/–^ mice exhibited decreased miR-9 expression in the aortic lysate (*p* < 0.05; Figure 4B). These findings demonstrated that miR-9 was poorly-expressed in ACS.

### Up-Regulation of miR-9 Suppresses Atherosclerosis *via* Inhibition of SDC2-Dependent FAK/ERK Signaling Pathway Activation in HFD-Fed Mice

**FIGURE 5:**
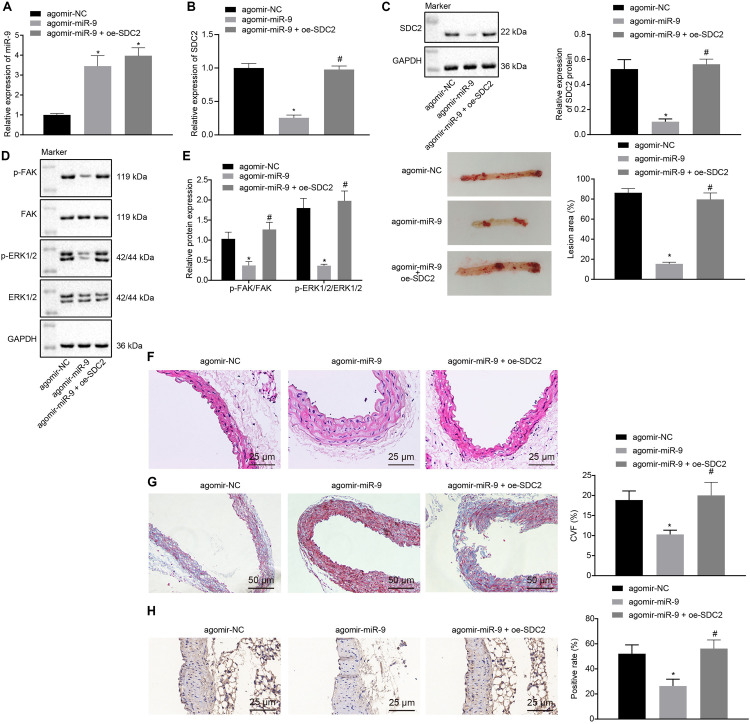
miR-9 relieves atherosclerosis by disrupting the SDC2-dependent FAK/ERK signaling pathway in mice. **(A)** miR-9 expression patterns determined by RT-qPCR in the aortic lysate of mice treated with miR-9 agomir, normalized to U6; **(B)** SDC2 mRNA expression patterns determined by RT-qPCR in the aortic lysate of mice treated with miR-9 agomir or in combination with oe-SDC2, normalized to GAPDH; **(C)** Representative Western blots of SDC2 protein and its quantitation in the aortic lysate of mice treated with miR-9 agomir or in combination with oe-SDC2, normalized to GAPDH; **(D)** Representative Western blots of FAK, and ERK1/2 proteins and their quantitation in the aortic lysate of mice treated with miR-9 agomir or in combination with oe-SDC2, normalized to GAPDH; **(E)** Aortic plaque area measured using oil red O staining in mice treated with miR-9 agomir or in combination with oe-SDC2; **(F)** The lesion degree of aorta determined using HE staining in mice treated with miR-9 agomir or in combination with oe-SDC2 (400×); **(G)** The proliferation of collagen fibers determined using Masson’s trichrome staining in aorta of mice treated with miR-9 agomir or in combination with oe-SDC2 (200×); and **(H)** Mac-3-labeled macrophages surrounding the aorta were measured using immunohistochemistry in mice treated with miR-9 agomir or in combination with oe-SDC2 (400×); *p < 0.05 *vs.* mice treated with agomir NC; *n* = 8 for mice upon each treatment. All data were measurement data and expressed as mean ± standard deviation. Comparisons were analyzed using the unpaired *t*-test. The experiment was repeated 3 times.

Upon uncovering poorly-expressed miR-9 in ACS, we speculated that the elevation of miR-9 could ameliorate atherosclerosis. The results of RT-qPCR displayed that the expression of miR-9 was up-regulated in the aortic lysate of miR-9 agomir-treated mice (Figure 5A). Compared to mice treated with agomir NC, mice treated with miR-9 agomir showed decreased SDC2 expression, while dual treatment with miR-9 agomir and oe-SDC2 brought about the opposite results (*p* < 0.05; Figure 5B). The expression patterns of SDC2, p-FAK/FAK, and p-ERK/ERK detected in the aortic lysate of mice with Western blot analysis were also found to be all significantly reduced upon treatment with miR-9 agomir compared to mice treated with agomir NC, while these affects were negated following co-treatment with miR-9 agomir and oe-SDC2 (all *p* < 0.05; Figures 5C,D). Oil red O staining revealed that compared with mice treated with agomir NC, the mice treated with miR-9 agomir had reduced aortic plaque area, which was neutralized following co-treatment with miR-9 agomir and oe-SDC2 (*p* < 0.05; Figure 5E). Additionally, HE staining illustrated hyperplasia, disordered arrangement and partial loss of endothelial cells in mice treated with agomir NC. When compared to mice treated with agomir NC, the lesion degree of mice treated with miR-9 agomir was also found to be relieved. However, the aortic lesion degree was aggravated in mice upon treatment with both miR-9 agomir and oe-SDC2 (*p* < 0.05; Figure 5F). The indicators of VR revealed that compared to mice treated with agomir NC, mice treated with miR-9 mimic exhibited decreased MT and MT/LD and increased LD, whereas treatment with both miR-9 agomir and oe-SDC2 brought about the opposite results (all *p* < 0.05; Table 3). Findings of Masson’s trichrome staining, immunohistochemistry, and ELISA displayed that compared with mice treated with agomir NC, mice treated with miR-9 agomir presented with reduced proliferation of collagen fibers, vascular CVF, macrophages (labeled by Mac-3 antibody surrounding the aorta of mice) in atherosclerotic plaque of aorta, and levels of serum IL-6, IL-1β, and TNF-α, which could all be counteracted by dual treatment with miR-9 agomir and oe-SDC2 (all *p* < 0.05; Figures 5G,H and Table 4). The abovementioned data indicated that the elevated miR-9 ameliorated atherosclerosis by inhibiting SDC2-dependent FAK/ERK signaling pathway activation in HFD-fed mice.

**TABLE 3:** Comparisons of VR indicators in mice treated with miR-9 agomir or in combination with oe-SDC2.

Groups	MT	LD	MT/LD (%)
agomir NC	70.05 ± 10.02	2.91 ± 0.56	24.69 ± 5.41
miR-9 agomir	34.91 ± 2.79*	7.13 ± 0.28*	4.90 ± 0.43*
miR-9 agomir + oe-SDC2	68.26 ± 9.62	3.12 ± 0.25	23.76 ± 4.45

miR-9, microRNA-9; VR, vascular remodeling; MT, medium wall thickness; LD, lumen diameter; MT/LD, medium wall thickness/lumen diameter; NC, negative control; *p < 0.05 *vs.* mice treated with agomir NC; *n* = 8 for mice upon each treatment; All data were measurement data and expressed as mean ± standard deviation. Comparisons were analyzed using the unpaired *t*-test. The experiment was repeated 3 times.

**TABLE 4:** Levels of serum inflammatory factors of mice treated with miR-9 agomir or in combination with oe-SDC2.

Inflammatory factors (pg/mL)	IL-6 (pg/mL)	IL-1β (pg/mL)	TNF-α (pg/mL)
agomir NC	6.89 ± 1.16	12.27 ± 2.48	8.04 ± 0.95
miR-9 agomir	3.50 ± 0.29*	6.12 ± 0.31*	4.33 ± 0.38*
miR-9 agomir + oe-SDC2	6.56 ± 1.05	12.76 ± 2.62	7.86 ± 0.82

IL, interleukin; TNF-α, tumor necrosis factor-α; NC, negative control; *p < 0.05 *vs.* mice treated with agomir NC; *n* = 8 for mice upon each treatment; All data were measurement data and expressed as mean ± standard deviation. Comparisons were analyzed using the unpaired *t*-test. The experiment was repeated 3 times.

## Discussion

Atherosclerosis plays a critical pathological role in the majority of patients with ACS (Waterbury et al., 2020). A recent study has highlighted the vital roles played by miRNAs in the pathogenesis of atherosclerosis (Skuratovskaia et al., 2019), while the cellular mechanism of miR-9 underlying atherosclerosis development in ACS is still poorly understood. Thus, the current study set out to explore the possible effects of miR-9 on atherosclerosis in ACS. Taken together, our data revealed that the up-regulation of miR-9 could potentially relieve atherosclerosis in ACS by down-regulating SDC2 *via* inhibition of the FAK/ERK signaling pathway.

Initially, findings from the current study revealed that miR-9 was poorly-expressed and SDC2 was highly-expressed in atherosclerosis. Interestingly, a prior study has investigated the aberrant expressions of miRNAs in ACS, providing novel therapeutic targets for the deadly syndrome (Wei et al., 2016). Adding to this, dysregulation of miRNA expression has also been proven to be related to a variety of cardiac diseases, including atherosclerotic plaque formation (Shirvani Samani and Meder, 2016). Moreover, significantly diminished expression of miRNAs such as miR-17, miR-126, and miR-155, has been previously documented in patients with coronary artery disease (Izawa and Amano, 2015). Besides that, Yu et al. (2020) observed poor miR-9 expression in n the aortic tissue of mice with atherosclerosis. miRNAs possess the ability to modulate gene expression post-transcriptionally by comprehensively interacting with the 3’UTR of specific target mRNAs (Ivey and Srivastava, 2015). In the present study, the bioinformatics website in combination with dual-luciferase reporter gene assay validated that SDC2 was a potential target gene of miR-9, where miR-9 could negatively-regulate the SDC2 expression. Geeraert et al. (2007) have revealed that significantly up-regulated mRNA and protein levels of SDC2 were noted in the process of atherosclerotic plaque formation, which was in line with our results. All these evidences support the idea behind miR-9 down-regulation and SDC2 up-regulation in aortic tissues, while miR-9 shared a negative-correlation with SDC2.

Additionally, our data demonstrated that up-regulation of miR-9 reduced the aortic plaque area, the proliferation of collagen fibers, Mac-3-labeled macrophages as well as the levels of IL-6, IL-1β, and TNF-α by down-regulating SDC2 *via* inhibition of the FAK/ERK signaling pathway, thereby ameliorating atherosclerosis in ACS. Advanced atherosclerotic changes accompanied by concomitant thrombus formation are one of the major characteristics of ACS progression (Libby et al., 2019). Meanwhile, miRNAs has been documented to correlate to atherosclerotic changes. For instance, miR-100 expression in the aorta is negatively-correlated with the percentage of fibrous volume in coronary plaques (Izawa and Amano, 2015). Additionally, miR-100 has also been found to be overexpressed in unstable human plaques (Cipollone et al., 2011; Maitrias et al., 2015). Existing literature further implicates miR-150 with endothelial apoptosis, which implies a potential therapeutic target for endothelial dysfunction and atherosclerosis (Qin et al., 2017). A recent study has reported that the over-expression of miR-150 maintains the function of endothelial cells and suppresses VR by inhibiting pentraxin-3 (PTX3) expression, as well as activation of the NF-κB signaling pathway in ACS mice (Luo et al., 2018). Moreover, up-regulated miR-9 is known to suppress the formation of vulnerable atherosclerotic plaques and enhance VR in ACS mice through negative-regulation of the p38MAPK pathway *via* OLR1, while our findings indicate a similar function via inhibition of the FAK/ERK signaling pathway (Yu et al., 2020). Over-expression of miR-9 in bone marrow-derived mesenchymal stem cells is also known to diminish the levels of IL-6, IL-1β, and TNF-α in acute pancreatitis, highlighting the critical role of miR-9 up-regulation in inhibition of inflammatory responses (Qian et al., 2017). Similarly, miR-9 was involved in suppression of inflammatory responses in atherosclerosis, wherein miR-9 inhibited the activation of the NLRP3 inflammasome, and thus attenuated atherosclerosis-related inflammation through the JAK1/STAT signaling pathway (Wang et al., 2017). Moreover, the SDC2 gene plays a key role in the regulation of the TGF-β signaling pathway as a co-receptor for growth factors (Huang et al., 2009). Another study also documented the elevation of SDC2 during atherosclerotic plaque formation (Geeraert et al., 2007), suggesting that inhibition of SDC2 could suppress the process of atherosclerotic plaque formation, which is very much in line with our findings. Captivatingly, SDC2 can also augment the activation of the FAK/ERK signaling pathway (Jang et al., 2017), while SDC2 depletion results in the inactivation of the FAK/ERK signaling pathway. Activation of FAK and ERK1/2 signaling pathways has been suggested to promote angiogenesis in ApoE^–/–^ mice (Hu et al., 2018). Furthermore, Zhang et al. (2012) found that the activated ERK2 signaling pathway was implicated in the development of ACS, adding more substance to the theory behind that inhibition of the ERK2 signaling pathway could suppress the progression of ACS. Overexpression of miR-9 can suppress the expression of p-AKT, and p-ERK in colorectal cancer HCT116 cells (Park et al., 2019). Furthermore, the activation of the FAK/AKT signaling pathway has been reported to be inhibited by the increased miR-9 levels in ovarian serous carcinoma cells (Tang et al., 2013). The abovementioned findings suggested that elevated miR-9 could relieve atherosclerosis by decreasing SDC2 expression *via* suppression of the FAK/ERK signaling pathway.

## Conclusion

In conclusion, the current study uncovered evidence that miR-9 could potentially decelerate the occurrence and progression of atherosclerosis in ACS. Besides, miR-9 may serve as a negative regulator of SDC2 to suppress atherosclerosis in ACS through inhibition of the FAK/ERK signaling pathway. Thus, miR-9 may serve as a potential therapeutic target for the treatment of atherosclerosis in patients with ACS. However, cellular sources remained elusive of SDC2 and miR-9 in the arterial wall, and the hypothesis of endothelial nature was not assessed, which should be elucidated in future investigations.

## Data Availability Statement

The raw data supporting the conclusions of this article will be made available by the authors, without undue reservation, to any qualified researcher.

## Ethics Statement

Written informed consents were obtained from all patients prior to the study. The protocols of this study were approved by the Ethics Committees of the Fourth Affiliated Hospital of Harbin Medical University and strictly in accordance with the ethical principles for medical research involving human subjects of the Helsinki Declaration. Animal experiments were performed strictly in accordance with the Guide to the Management and Use of Laboratory Animals issued by the National Institutes of Health. The protocol of animal experiments was approved by the Institutional Animal Care and Use Committee of the Fourth Affiliated Hospital of Harbin Medical University.

## Author Contributions

RZ designed the study. BS collated the data, XH and ZS analyzed and produced the initial draft of the manuscript. LS and SW contributed to drafting the manuscript. All authors have read and approved the final submitted manuscript.

## Conflict of Interest

The authors declare that the research was conducted in the absence of any commercial or financial relationships that could be construed as a potential conflict of interest.

## Footnotes

1 https://www.ncbi.nlm.nih.gov/geo/

2 http://master.bioconductor.org/packages/release/bioc/html/limma.html

3 https://cran.r-project.org/web/packages/pheatmap/index.html

4 http://diana.imis.athena-innovation.gr/DianaTools/index.php?r=microT_CDS/index

5 http://carolina.imis.athena-innovation.gr/diana_tools/web/index.php?r=tarbasev8%2Findex

6 http://www.targetscan.org/vert_71/

7 http://www.microrna.org/microrna/home.do
