# Supplementary material for: microRNA-9 Inhibits Vulnerable Plaque Formation and Vascular Remodeling via Suppression of the SDC2-Dependent FAK/ERK Signaling Pathway in Mice With Atherosclerosis
Source: Front Physiol. 2020 Jul 16;11:804. doi: 10.3389/fphys.2020.00804 (PMC7378740; doi:10.3389/fphys.2020.00804)
Supplement: Supplementary file 1 [file Table_1.DOCX]

**Supplementary Table 1** Primer sequences for RT-qPCR

| Target | Sequence (5’-3’) |
| --- | --- |
| miR-9 | Forward: ACACTCCAGCTGGGAGTATGTCGATCTATTG |
|  | Reverse: TGGTGTCGTGGAGTCG |
| SDC2 | Forward: ACACTCCAGCTGGGAGTATGTCGATCTATTG |
|  | Reverse: TCGTGGAGTCG |
| U6 | Forward: CTCGCTTCGGCAGCACA |
|  | Reverse: AACGCTTCACGAATTTGCGT |
| GAPDH | Forward: AGCCACATCGCTCAGACA |
|  | Reverse: GCCCAATACGACCAAATAA |
